# The Multidirectional Biological Activity of Resveratrol: Molecular Mechanisms, Systemic Effects and Therapeutic Potential—A Review

**DOI:** 10.3390/nu18020313

**Published:** 2026-01-19

**Authors:** Łukasz Kogut, Czesław Puchalski, Danuta Katryńska, Grzegorz Zaguła

**Affiliations:** 1 Department of Bioenergetics, Food Analysis and Microbiology, Institute of Food Technology and Nutrition, Faculty of Technology and Life Science, University of Rzeszów, Aleja Rejtana 16C, 35-959 Rzeszów, Poland; lukasz.kogut2@wp.pl (Ł.K.); cpuchal@ur.edu.pl (C.P.); 2 Department of Zoology, Institute of Zoology and Earth Sciences, University of the National Education Commission in Krakow, 30-084 Krakow, Poland; dkatrynska@danlab.pl

**Keywords:** resveratrol, polyphenols, inflammation, oxidative stress, SIRT1, AMPK, hepatoprotection, neuroprotection, gut microbiota

## Abstract

**Background/Objectives:** Resveratrol is a multi-target polyphenolic stilbene widely studied for its antioxidant, anti-inflammatory, cardioprotective, hepatoprotective, neuroprotective, immunomodulatory and anticancer properties. This review summarizes current evidence on its molecular mechanisms, therapeutic potential, metabolic interactions and biological implications, with particular emphasis on bioavailability, signaling pathways and organ-specific actions. **Methods:** A comprehensive literature review was conducted focusing on recent in vitro, in vivo and clinical studies evaluating resveratrol’s biochemical activity, molecular targets and physiological effects. Special attention was given to oxidative stress regulation, inflammatory signaling, mitochondrial function, metabolic pathways, gut microbiota interactions, and its influence on chronic diseases. **Results:** Resveratrol modulates several key signaling pathways including NF-κB, SIRT1, AMPK, MAPK, Nrf2 and PI3K/AKT/mTOR. It reduces oxidative stress, inhibits inflammatory cytokines, regulates apoptosis, improves mitochondrial performance, and activates endogenous antioxidant systems. The compound demonstrates protective effects in cardiovascular diseases, hepatic steatosis, neurodegenerative disorders, metabolic dysfunction, and various cancers through anti-inflammatory, anti-proliferative and anti-fibrotic mechanisms. Additionally, resveratrol beneficially alters gut microbiota composition and microbial metabolites, contributing to improved metabolic homeostasis. Despite high intestinal absorption, systemic bioavailability remains low; however, novel nanoformulations significantly enhance its stability and plasma concentrations. **Conclusions:** Resveratrol exhibits broad therapeutic potential driven by its capacity to regulate oxidative, inflammatory, metabolic and apoptotic pathways at multiple levels. Its pleiotropic activity makes it a promising candidate for prevention and complementary treatment of chronic diseases. Advances in delivery systems and microbiota-derived metabolites may further enhance its clinical applicability.

## 1. Introduction

Resveratrol (3,5,4′-trihydroxystilbene) is a natural polyphenolic compound belonging to the stilbene subgroup, also classified as a phytoalexin [1,2,3,4]. From a chemical point of view, the structure of the compound comprises two phenyl rings connected by a two-carbon bridge [2,3,4,5]. Its antioxidant properties result from the presence of compressed double bonds and hydroxyl groups [3]. It occurs in two geometric isomers: trans and cis forms [1,4,5,6]. Under natural conditions, the trans isomer form predominates, characterised by significantly higher biological activity [1,3,4,5,7]. It can also undergo isomerisation to the cis form under the influence of an alkaline environment, UV light and high temperature [1,3,5,8]. Among stilbene derivatives, piced, a resveratrol glycoside, and trans-ε-winiferin, a resveratrol dimer, are particularly noteworthy. Both compounds have high application potential in cosmetology due to their strong stimulation of sirtuin 1 activity, exhibiting skin whitening and discolouration reduction effects [6,9]. Plants such as grapevines (*Vitis vinifera*) synthesise resveratrol in response to stress factors, including pathogenic infections, mechanical damage and exposure to UV radiation or ozone. which is a consequence of genetically determined activation of stilbene synthase genes. This compound acts as a phytoalexin, limiting the growth of pathogens and protecting cells from oxidative stress, and when consumed by other organisms, it has the ability to modulate the expression of genes associated with protective mechanisms and the regulation of inflammatory processes [3,4,5,6].

Despite its broad biological activity, the use of resveratrol is limited due to its poor bioavailability and low solubility. It is a lipophilic substance with good solubility in organic solvents such as ethanol [4,8,10]. Nevertheless, its absorption by the human body remains low. Current research mainly focuses on methods of increasing bioavailability by combining resveratrol with piperine from black pepper, which inhibits liver metabolism and increases absorption, as well as the use of lipid nanocarriers. This is particularly important in dermatological applications, where the liposomal or nanoemulsion form allows for increased penetration through the skin barrier [1,3,5]. Despite the existing problem of low bioavailability, resveratrol is a safe compound. No significant adverse effects have been detected in humans after oral administration of high doses (5 g per 70 kg of body weight), which indicates its usefulness in supplementation and supportive therapy [3,5].

The importance of resveratrol in medicine and nutrition stems primarily from its ability to modulate many key physiological processes that play a fundamental role in the pathogenesis of chronic diseases. Numerous studies have confirmed its involvement in the regulation of inflammatory responses, oxidative stress and mitochondrial function, which translates into broad protective effects in the cardiovascular, nervous, metabolic and hepatic systems. Its role as a modulator of pathways responsible for cellular homeostasis is particularly important, making it a compound with high preventive potential in the context of age-related diseases [11,12,13,14,15,16,17,18,19,20,21,22,23,24,25,26,27,28,29,30,31,32,33,34,35,36,37,38].

Resveratrol exhibits multidirectional activity due to its influence on a number of signalling pathways responsible for controlling inflammatory, metabolic and proliferative processes. Its ability to activate SIRT1, AMPK and Nrf2, as well as inhibit the NF-κB pathway, has been documented, leading to a reduction in the expression of pro-inflammatory cytokines, improved mitochondrial function and reduced oxidative stress. As a result, resveratrol modulates key mechanisms responsible for the development of pathologies such as atherosclerosis, insulin resistance, neurodegeneration and chronic liver disease [11,12,13,14,15,16,17,18,19,24,25,26,27,28,29,30,31,32,33,34,38,39,40,41,42,43,44,45,46,47,48,49,50,51].

The aim of this paper is to discuss in detail the biological properties of resveratrol in the context of its key molecular pathways, anti-inflammatory, antioxidant, metabolic, and immunomodulatory effects. Current data on its impact on the gut microbiota and potential applications in the prevention and treatment of chronic diseases are also considered. The paper also emphasizes the importance of modern strategies to increase the effectiveness of resveratrol in the human body.

## 2. Materials and Methods

The literature included in this review was identified through a structured search of PubMed/MEDLINE, Scopus, Embase and Google Scholar. The search strategy focused on original research papers and review articles published between 2000 and 2025, with particular emphasis on the last decade. The following combinations of keywords were used together with Boolean operators (“AND”, “OR”): *resveratrol*, *trans-resveratrol*, *stilbenes*, *polyphenols*, *oxidative stress*, *inflammation*, *NF-κB*, *SIRT1*, *AMPK*, *gut microbiota*, *hepatoprotection*, *cardioprotection*, *neuroprotection*, *metabolic syndrome*, *NAFLD/MASLD*, *cancer*, and *immune modulation*. Additional sources were identified through manual screening of reference lists.

Studies were included if they examined resveratrol directly and provided relevant information on its biological activity, molecular mechanisms, pharmacokinetics, or health-related effects. Eligible papers encompassed in vitro, in vivo and human studies written in English and available in full text. Publications were excluded if they lacked mechanistic relevance, focused on unrelated polyphenols, presented insufficient methodological quality, or were limited to editorials, conference abstracts, commentaries, or non–peer-reviewed sources. Duplicate records were removed during the selection process.

All retrieved articles were screened independently to ensure scientific relevance and coherence with the aims of this review. Extracted data included molecular pathways influenced by resveratrol, biological outcomes, dose-dependent effects and interactions with metabolic and inflammatory processes. As this work is a narrative review, no ethical approval or study registration was required.

## 3. Sources and Methods of Acquisition

The main and most important dietary source of resveratrol is grapes and wine products such as red wines, white wines and rosé wines [52]. The highest amounts of this compound are found in the skins of dark varieties of Vitis vinifera. It is obtained from here by extraction with organic solvents or enzymatic hydrolysis of glycosides. The amount of resveratrol in fresh skins ranges from 50 to 100 µg/g, and this amount may increase under stressful conditions [53,54]. Red wines macerated with skins usually contain between 0.2 and 7 mg/L of total resveratrol, and in some reported cases this value reaches as high as approx. 15 mg/L. In this case, resveratrol is released during prolonged contact between fermenting must and skins and seeds [54,55]. For comparison, the amount of resveratrol in white wines does not usually exceed 1 mg/L, due to the short contact time between the skins and the must or the complete lack of contact [54]. In grape juices, the content of the compound ranges from about 4 to 7 mg/L, while in concentrates, due to the removal of water, this value increases to about 12–21 mg/L. Resveratrol is obtained by direct pressing of the fruit, sometimes assisted by ultrasound [56].

Apart from grapes, there are several other important sources of resveratrol. Peanuts contain on average 3 to 10 µg/g of resveratrol in the kernel, while in the shell these values are significantly higher, reaching several dozen µg/g. It is obtained by extracting crushed material with methanol or ethyl acetate at elevated temperatures [57,58,59,60]. Among berries, mulberries, especially the black variety (*Morus nigra*), are particularly noteworthy, with values ranging from 20 to 60 µg/g. In the case of berries, it is obtained by solvent extraction and isolation of the polyphenol fraction using HPLC [53,55]. In walnuts, the resveratrol content reaches 1585 µg/100 g, making it one of the best sources among nuts. It is obtained using a method of extraction with alcohol and water [61]. Soybeans are also an additional, albeit relatively moderate, source of this compound. They contain approximately 10.75 µg/100 g. Resveratrol can be extracted from soybeans by using ethanol extraction with water from finely ground seeds. Although the content is low, soybean consumption in the human diet is relatively high, which may further increase the supply of resveratrol to the human body [58,59,61]. This compound is also found in many berries, but its content depends on factors such as climate, variety, and type of cultivation. The highest amounts were found in mandarins (approx. 1061 µg/100 g), sweet potatoes (>900 µg/100 g) and peaches (approx. 460 µg/100 g). In this group, resveratrol is obtained by extracting the pulp and peel tissues with polar solvents or by minimising exposure to oxygen and light [61]. In raspberries, blueberries, currants, strawberries, and apples, the resveratrol content ranged from 0.3 to 2 mg/kg. For these fruits, resveratrol can be extracted in several ways, i.e., by cold pressing, centrifugation, freeze-drying or under conditions that minimise oxidation [62,63]. In turn, the resveratrol content in cherry tomatoes can reach up to approx. 19 mg/g (the highest amounts are found in the skin) [58,63,64]. In coffee products, the trans-resveratrol content is relatively low but stable. In natural cocoa, it averages about 11.4 µg/kg, in alkalized cocoa about 13.5 µg/kg, and in dark chocolate (55% cocoa content) about 7.5 µg/kg. Here, the process of obtaining resveratrol differs from other examples, i.e., during the fermentation of cocoa beans, their roasting and pressing of cocoa mass [65].

## 4. Bioavailability and Absorption of Resveratrol

Resveratrol is characterized by a paradoxical relationship between absorption and bioavailability [66]. Despite this fact, its absorption in the gastrointestinal tract is quite high, reaching 75% [67,68], while its systemic bioavailability in humans is less than 1%. This means that only a small dose of the substance reaches our bloodstream unchanged. The absorption process occurs mainly through transepithelial diffusion. Further stages of metabolism in the small intestine and liver effectively reduce the amount of biologically active compound [66,67,69,70,71,72]. The relatively low bioavailability is due to adverse physicochemical changes and intensive pre-systemic and systemic metabolism [66,67].

The most limiting factor is its relatively low solubility in water, which is due to the fact that resveratrol belongs to a group of lipophilic compounds with a log *p* value of 3.1 and is classified as class II in the Biopharmaceutical Classification System (BCS). It is characterized by high permeability but low water solubility (approx. 0.13 mM). This makes it a virtually insoluble substance [66,73]. The second important limiting factor is its high sensitivity and low chemical stability when exposed to light. This causes photodegradation and a drastic reduction in the activity of the compound [66,68,72,73]. Another key aspect is the intense first-pass metabolism, which occurs mainly in the intestines and liver [68,69]. When administered orally, resveratrol is rapidly converted into conjugated forms, mainly glucuronide and sulfates [66,68,74,75]. Studies clearly show that free resveratrol in the body accounts for only 4.4% of total exposure, while its sulfates account for as much as 93.1% [74]. The intestinal microflora then metabolizes resveratrol, leading to the formation of reduced forms of the compound, such as dihydroresveratrol, whose conjugates and other polar metabolites can account for up to 50% of the consumed dose [68,76].

The bioavailability of resveratrol is also influenced by ABC transport proteins, such as MRP3 (Multidrug Resistance-associated Protein 3) and BCRP (Breast Cancer Resistance Protein), which participate in its excretion and limit its distribution to tissues [76,77]. Furthermore, administration of resveratrol in its classic powder or capsule form results in a short-term and slight increase in its concentration in the blood, which is insufficient to achieve a therapeutic effect [68,74,76].

To overcome the problem of low bioavailability of resveratrol, a number of innovative methods have been developed to improve its solubility, stability, and reduce its intensive metabolism. Nanotechnology is one of the most effective ways to improve the bioavailability of resveratrol [67,72,73]. The use of nanocarriers has increased its solubility in an aqueous environment, increased protection against light degradation, and prolonged its presence in the bloodstream. The use of soluble lipid formulations mimicking natural plant resveratrol in human studies has resulted in an 8.8-fold increase in plasma resveratrol levels compared to the traditional powder form. The method involves coating resveratrol particles with a lipid coating, which reduces their hydrophobicity and prevents the crystals from sticking together, allowing for better dissolution in water and increased absorption in the intestine [74,78,79]. A similar situation was observed with the use of LipiSperse^®^ technology. The method focuses on increasing the dispersion of lipophilic components, which in turn allows for a 2-fold increase in the area under the concentration-time curve (AUC) and a 3-fold increase in the maximum plasma concentration (Cmax) of trans-resveratrol conjugates after oral administration of 150 mg of Veri-Sperse^®^ compared to the classic powder. The method involves surrounding resveratrol crystals with lipids, which reduce their hydrophobicity and prevent agglomeration, making the compound easier to disperse in the intestinal contents and more effectively absorbed by the intestinal epithelium. Clinical trials also monitored the safety of this technology; no serious adverse events were reported with a single dose of 150 mg, although at very high doses of resveratrol (above 2.5 g) the risk of side effects increases, indicating the need for careful dosing [75].

When using nanoparticle systems, casein nanoparticles (Rsv-NP-C) are particularly effective, increasing the oral bioavailability of resveratrol tenfold (26.5%) in rats, allowing the substance to remain in the plasma for up to 8 h. Despite its high efficacy, it is necessary to monitor potential effects on the immune system, liver and spleen. This mechanism is related to lymphatic transport, which completely bypasses pre-systemic metabolism. This method involves creating resveratrol complexes with casein, which facilitate penetration through the intestinal mucus layer and enable gradual release of the compound at the intestinal epithelium, prolonging its presence in the circulation [80]. Much better results were obtained with zein nanoparticles, which improved the bioavailability of resveratrol 19.2-fold (50%) compared to the PEG400:water hydrophilic-polymer system. Zeina, as a hydrophobic carrier, protects resveratrol from degradation and facilitates transport across cell membranes, increasing bioavailability. Although zein nanoforms are considered safe (food-grade), it is still recommended to evaluate their interaction with biological barriers and the immune system prior to clinical application [81].

Beneficial effects were also observed with the use of solid lipid nanoparticles (SLNs), which extend the half-life of the compound from 2.4 h for free resveratrol to 11.5 h. In addition, this technology enables 8-fold better absorption when administered orally. The authors emphasise that SLNs protect cells from the cytotoxic effects of resveratrol, but require further safety assessment for long-term use [71,81]. Their ability to better penetrate the blood-brain barrier has also been documented (17.3 ± 0.6 mg/g in brain tissue compared to 3.5 ± 0.4 mg/g of free resveratrol. Resveratrol embedded in a solid lipid matrix is released slowly, which protects it from rapid metabolism and allows it to reach tissues such as the brain [72]. Nanosuspensions, in turn, significantly increased pharmacokinetic parameters by 1.3 times for AUC and 3.4 times for Cmax. In this case, resveratrol in the form of nanocrystals has an increased dissolution surface area and is absorbed more quickly in the intestine; intranasal administration allows direct transport to the brain [72,77].

Another group of effective carriers are cyclodextrins (CDs), which form inclusion complexes, significantly improving the solubility of resveratrol [72,81] by 33 to 48 times in the case of β-CD-based nanosponges, as well as its photostability. The authors emphasise that unmodified β-cyclodextrin may exhibit nephrotoxicity; therefore, safe forms such as HP-β-CD are preferred. Cyclodextrin-based nanosponges may exhibit increased cytotoxicity in some cell models, which is a clinical limitation of these carriers [66]. The JOTROL™ micellar formulation (10% resveratrol) also shows interesting results in terms of bioavailability. Oral administration of a single 500 mg dose resulted in a high Cmax of 455 ng/mL, while administration of 1 g of standard capsules resulted in a Cmax of only 85 ng/mL. Resveratrol in micelles is absorbed by the lymphatic system, bypassing liver metabolism, which allows for higher plasma concentrations to be achieved. In clinical trials, participants were monitored for adverse events; only mild symptoms such as drowsiness (29.2%) and headache (20.8%) were reported, highlighting the good tolerability of the micellar formulation [70].

Another approach to increasing the bioavailability of resveratrol is co-administration with metabolism inhibitors such as piperine, an alkaloid derived from black pepper (*Piper nigrum*). In vivo studies conducted on mice have shown that co-administration of piperine significantly reduces the glucuronidation of resveratrol, resulting in a 1544% increase in maximum concentration (Cmax) and a 229% increase in area under the curve (AUC) compared to pure resveratrol. This study demonstrates the efficacy of piperine, but also of similar compounds, as pharmacokinetic enhancers of resveratrol. Despite promising results in animal models, piperine did not increase the bioavailability of resveratrol in clinical trials. Furthermore, it requires monitoring for potential interactions with other drugs, as it inhibits enzymes that metabolise medicinal substances [66,78].

The last important strategy for increasing the bioavailability of resveratrol is to search for and use chemical analogues with better stability and bioavailability. The most popular substance of this type is pterostilbene, a dimethyl ether analogue of resveratrol (3,5-dimethoxy-4′-hydroxy-trans-stilbene) [67,73]. Thanks to its significantly increased lipophilicity and easier penetration through cell membranes, this compound has 3–4 times higher oral bioavailability and higher total plasma concentration than resveratrol [66,67,73,75]. Another noteworthy treatment is the combination of resveratrol with methoxyl groups, which is an effective method of improving its pharmacokinetic properties [73]. An example of such a treatment is trans-3,4,5,4′-tetramethoxystilbene (DMU 212), a compound that exhibits a significantly more favorable distribution profile, achieving higher concentrations in the small intestine, colonic mucosa, and brain tissue compared to classic resveratrol [73].

## 5. Mechanism of Action, Potential Health Benefits, and Therapeutic Applications

### 5.1. Anti-Inflammatory Effect

Inhibition of the nuclear factor kappa B (NF-κB) pathway is one of the best-known anti-inflammatory mechanisms induced by resveratrol. This factor acts as a central regulator of the transcription of pro-inflammatory genes such as tumor necrosis factor alpha (TNF-α), interleukin 1 beta (IL-1β) and interleukin 6 (IL-6) [11,12,13,14,15,16]. In inflammatory conditions, IκB kinase (NF-κB inhibitor kinase) is activated, which phosphorylates the IκBα protein (NF-κB inhibitor), leading to its degradation. The released NF-κB complex containing the p56 subunit moves to the cell nucleus, where it activates the expression of pro-inflammatory genes [11,15]. Resveratrol inhibits the phosphorylation of both IκBα and IKK kinase (IκB kinase complex), which prevents the translocation of NF-κB to the nucleus, effectively suppressing the inflammatory cascade at the transcriptional level [12,13,15,16].

Another important aspect of resveratrol’s action is the modulation of NLRP3 inflammasome activity (a pyrin domain receptor in the NOD-like family), i.e., a multiprotein complex responsible for the processing and secretion of the pro-inflammatory cytokine interleukin 1 beta (IL-1β) [12,16]. Studies conducted on RAW 264.7 and J774A.1 macrophage lines have clearly shown that resveratrol significantly reduces IL-1β secretion both at rest and after stimulation with lipopolysaccharide (LPS), a classic inducer of inflammatory response. This mechanism involves the inhibition of NLRP3 and ASC (apoptosis-related protein containing a CARD domain) protein expression and the maintenance of mitochondrial integrity, which prevents inflammasome activation induced by factors such as LPS/adenosine triphosphate (ATP) or LPS/nigericin [12].

Resveratrol is a known activator of sirtuin 1 (SIRT1)—an enzyme from the class of histone deacetylases dependent on nicotinamide adenine dinucleotide (NAD^+^). Activation of SIRT1 leads to inhibition of the Toll-like receptor 4/nuclear factor kappa B/signal transducer and activator of transcription (TLR4/NF-κB/STAT) pathway, which reduces the production of inflammatory mediators in macrophages and mast cells [13,16,17,18]. In addition, SIRT1 activation limits oxidative stress and counteracts pro-inflammatory phenotypic changes in vascular endothelial cells, which is important in the context of preventing vasculitis and atherosclerosis [13].

Resveratrol also modulates the activity of mitogen-activated protein kinases (MAPKs), including JNK (c-Jun *N*-terminal kinase) and p38 MAPK [17,18]. These pathways are directly involved in regulating cellular responses to stress and inflammatory stimuli. By inhibiting their activity, resveratrol suppresses the expression of the AP-1 (activator protein 1) transcription factor, which interacts with NF-κB in the induction of inflammatory genes. Thanks to this action, resveratrol effectively reduces the expression of pro-inflammatory enzymes and cytokines, which contributes to both its anti-inflammatory and anti-cancer effects [16,19].

Resveratrol also directly affects the expression and secretion of many inflammatory mediators. Cell studies have shown that this compound lowers the levels of pro-inflammatory cytokines such as TNF-α, IL-1β, and IL-6 by inhibiting the transcription of their genes in lipopolysaccharide-stimulated microglia and macrophages (LPS) [13,15,16,17,19,20]. At the same time, it can increase the expression of anti-inflammatory interleukin 10 (IL-10), which activates the Janus kinase 1/signal transducer and activator of transcription 3 (JAK1/STAT3) pathway and leads to the induction of cytokine signaling suppressor protein 3 (SOCS3)—a negative regulator of cytokine signaling [20]. Resveratrol also inhibits the activity of cyclooxygenase-1 (COX-1) and cyclooxygenase-2 (COX-2) enzymes that catalyze the synthesis of prostaglandin, especially prostaglandin E2 (PGE2), whose excessive production is associated with pain, fever, and swelling [14,16,17,21,22]. Another important mechanism of action is the reduction in inducible nitric oxide synthase (iNOS) expression and the reduction in nitric oxide (NO) overproduction, a factor toxic to tissues in a state of chronic inflammation [11,13,15,16,23]. In addition, resveratrol reduces the expression of adhesive molecules such as intercellular adhesion molecule (ICAM-1) and vascular cell adhesion molecule (VCAM-1), which inhibits leukocyte adhesion to the endothelium and limits the migration of inflammatory cells to sites of inflammation [19,22,23].

The anti-inflammatory properties of resveratrol have been confirmed in numerous in vitro laboratory and in vivo animal models covering various disease entities. In cardiovascular diseases, resveratrol inhibits vascular inflammation by blocking NF-κB activation in coronary artery endothelial cells and suppressing the Toll-like receptor 4/NF-κB pathway [13,17,23]. It also has a protective effect in ischemic-reperfusion injury of the heart muscle, limiting cytokine production and oxidative stress [13,19]. In the central nervous system, resveratrol alleviates neuroinflammation by reducing TNF-α, IL-1β, and NO through microglial activators. This action occurs, among other things, by inhibiting the Toll-like receptor 4/NF-κB/signal transduction and activation protein (TLR4/NF-κB/STAT) cascade, which is important in the context of neurodegenerative diseases such as Alzheimer’s disease [13,15,18]. In the respiratory system, resveratrol reduces lipopolysaccharide-induced lung damage (known as acute lung injury) by inhibiting the NLRP3 inflammasome and activating sirtuin 1 [12,13]. In chronic obstructive pulmonary disease (COPD), extracts rich in resveratrol limit cytokine secretion by alveolar macrophages [13,20]. This compound also exhibits anti-inflammatory effects in endometriosis, where it reduces the volume of endometrial lesions by inhibiting prostaglandin synthesis and reducing oxidative stress [14]. In inflammatory bowel diseases such as ulcerative colitis, resveratrol reduces inflammatory activity by inhibiting NF-κB activation, reducing TNF-α secretion, and limiting neutrophil recruitment [22,82]. Clinical studies have shown that resveratrol supplementation at a dose of 500 mg per day for six weeks in patients with ulcerative colitis led to a significant reduction in TNF-α levels, high-sensitivity C-reactive protein (hs-CRP) levels, and NF-κB activity in peripheral blood mononuclear cells (PBMCs) [82].

### 5.2. Impact on the Immune System and Immune Modulation

Resveratrol has strong immunomodulatory properties, which directly contribute to the homeostasis of the immune system. This compound regulates both innate and acquired immune responses by influencing the activity of T lymphocytes, macrophages, dendritic cells, and natural killer (NK) cells. Its action mainly consists of restoring the balance between pro- and anti-inflammatory reactions, which is particularly important in the context of the prevention and treatment of autoimmune diseases and cancer [83,84,85,86,87,88]. One of the main immunological mechanisms of resveratrol is the modulation of the balance between Th1 (type 1 helper T cells) and Th2 (type 2 helper T cells) lymphocyte subpopulations. Resveratrol inhibits Th1 activity and interferon γ (IFN-γ, interferon gamma) secretion, while promoting the Th2 profile associated with increased concentrations of IL-4 (interleukin 4) and IL-10 (interleukin 10). This action results in a shift in the response towards anti-inflammatory [85]. At the same time, resveratrol restores the balance between Th17 cells (type 17 helper T cells) and Treg (regulatory T cells) [85,87], increasing the expression of the transcription factor FOXP3 (forkhead box P3) and the number of Treg (CD4^+^CD25^+^Foxp3^+^) [83,85,87], while inhibiting the secretion of interleukin 17 (IL-17). This limits excessive immune activation and protects against the development of autoimmunity [85,87].

Resveratrol also regulates macrophage functions, promoting the transition from a pro-inflammatory phenotype to M1 (pro-inflammatory macrophages) to anti-inflammatory M2 (anti-inflammatory macrophages). This increases the expression of interleukin 10 (IL-10), transforming growth factor beta (TGF-β) and arginase-1 (Arg-1, arginase 1), while inhibiting the secretion of tumor necrosis factor α (TNF-α, tumor necrosis factor alpha), interleukin 6 (IL-6, interleukin 6) and inducible nitric oxide synthase (iNOS, inducible nitric oxide synthase). As a result, it supports the suppression of inflammatory processes and tissue repair [85,89]. Similarly, with regard to dendritic cells (DC), RSV inhibits their maturation and antigen presentation capacity, promoting the formation of immunosuppressive DCs that produce interleukin 10 (IL-10) and reduce T cell activation [86]. However, it should be emphasised that most of the described effects concerning macrophage polarisation and dendritic cell function have been demonstrated mainly in in vitro studies and animal models, and their direct translation into clinical conditions requires further research involving humans [85,86,89].

This compound also demonstrates the ability to activate NK (natural killer) cells and CD8+ lymphocytes (cytotoxic T lymphocytes), increasing their cytotoxicity and interferon γ (IFN-γ) expression, which supports anti-tumor surveillance [84]. Resveratrol enhances NK activity at low concentrations, while inhibiting it at higher concentrations, indicating its biphasic nature [86]. In preclinical studies, resveratrol has shown protective effects in models of rheumatoid arthritis (RA) and systemic lupus erythematosus (SLE), alleviating the course of the disease and reducing exposure to proinflammatory cytokines [83,86]. However, it should be emphasised that most of the described immunomodulatory effects of resveratrol, including modulation of the Th1/Th2 axis and Th17/Treg balance, have been demonstrated mainly in in vitro studies and animal models. The authors of the cited studies point out that the immune response in humans is much more complex, and differences in physiology, resveratrol bioavailability and disease context may significantly influence the extent and direction of the observed effects. Therefore, these results should be treated as biologically relevant mechanistic signals requiring further verification in well-designed clinical trials [83,84,85,86,87,88,89].

### 5.3. Protection of the Cardiovascular System

The cardioprotective effect of resveratrol is multifaceted and involves several key mechanisms. The first of these is its antioxidant effect. Resveratrol neutralizes reactive oxygen species (ROS) such as hydroxyl radicals and superoxide anion radicals, thereby protecting endothelial cells and cardiomyocytes from oxidative stress [24,25,26,27,28]. In addition, it activates endogenous antioxidant enzymes such as superoxide dismutase (SOD), catalase (CAT), and glutathione peroxidase (GPx). These enzymes together form the body’s natural antioxidant defense system [25,29,30]. The activation of the Nrf2 transcription factor, which regulates the expression of genes encoding detoxification and antioxidant enzymes, plays a particularly important role here. This pathway is considered one of the key pathways in the prevention of oxidative damage to the heart muscle, including in the course of diabetic cardiomyopathy [24,28,29,31].

Another important aspect of resveratrol’s effect on the cardiovascular system is its impact on vascular endothelial function. This compound stimulates the expression and activity of endothelial nitric oxide synthase (eNOS), which translates into increased production of nitric oxide (NO), the main mediator of vasodilation. It significantly improves the bioavailability of NO, which translates into significantly better blood vessel relaxation, reduced peripheral resistance, and lower blood pressure [25,27,29,30,31,32,33]. In addition, this polyphenol counteracts the phenomenon of eNOS uncoupling, which under conditions of oxidative stress leads to the production of ROS instead of NO. This significantly improves the integrity and elasticity of blood vessels, inhibits the development of hypertension, and slows down the progression of atherosclerotic changes [29,31].

A significant element of resveratrol’s molecular action is the activation of sirtuin 1 (SIRT1), an NAD+-dependent protein deacetylase [24,28,29,31,32,33]. SIRT1 is considered one of the main mediators of the beneficial health effects of resveratrol, linking its action to the mechanisms of longevity and metabolic homeostasis. Deacetylation of eNOS by SIRT1 significantly increases the activity of this enzyme, supporting nitric oxide production and endothelial function [29,31,33]. At the same time, SIRT1 protects cardiomyocytes from oxidative stress-induced apoptosis, stabilizes energy metabolism, and reduces inflammation in the heart muscle. As a result, resveratrol not only has a protective effect, but also a regenerative one, supporting the proper functioning of heart and vascular cells [25,34].

The repeatedly proven anti-inflammatory effect of resveratrol plays an equally important role in the prevention of cardiovascular diseases. This compound inhibits the activation of the NF-κB transcription factor, which controls the expression of many pro-inflammatory genes [24,25,27,30,31,34,35]. As a result, it lowers the levels of cytokines such as TNF-α, IL-1β, and IL-6, reducing the chronic inflammation underlying atherosclerosis, hypertension, and ischemic heart damage. Resveratrol thus contributes to maintaining endothelial stability and preventing the deposition of atherosclerotic plaques [24,25,30,35].

Numerous preclinical studies have shown that resveratrol effectively lowers blood pressure and prevents cardiac hypertrophy. In rats with specific hypertension (SHR) and malignant hypertension (MHR), chronic resveratrol supplementation led to a significant decrease in blood pressure and reduced cardiac tissue damage [25,29,32,36,37]. This mechanism is associated with the inhibition of myeloperoxidase (MPO) activity, a reduction in transforming growth factor β (TGF-β) levels, and a decrease in myocardial cell apoptosis [37]. Despite the fact that human studies are inconclusive, meta-analyses indicate that high doses of resveratrol (≥300 mg/day) can significantly lower blood pressure [24,27].

The widely studied use of resveratrol in the context of anti-atherosclerotic activity is also noteworthy [27,33,35]. It inhibits the oxidation of LDL lipoprotein, a process considered key in the initiation of atherosclerotic changes. The oxidized form of LDL (LDL-ox) damages the endothelium and exacerbates inflammation in the vessel walls [25,26,27,32]. Resveratrol reduces the effects of these processes while also having a beneficial effect on the lipid profile by lowering LDL cholesterol and apolipoprotein B-100 (ApoB) [25,32]. This mechanism is associated with the activity of sirtuin 1 (SIRT1) and the inhibition of HMG-CoA reductase, a key enzyme in cholesterol biosynthesis. Although not all studies confirm an improvement in the classic lipid profile, positive changes in parameters related to cardiovascular risk have been observed [32,33]. However, it should be noted that despite the beneficial effects on LDL-ox, ApoB and molecular mechanisms, clinical data on the reduction in total LDL cholesterol and cardiovascular risk remain inconsistent, which is attributed, among other things, to the low bioavailability of resveratrol and the diversity of the study populations [25,26,27,32,33,35].

Research conducted in recent years has contributed to increased interest in resveratrol in the context of cardio-oncology, particularly cardiotoxicity caused by chemotherapeutic agents such as doxorubicin [26,36]. In studies conducted on animal models, the use of resveratrol significantly alleviated myocardial damage, while reducing oxidative stress, inhibiting cardiomyocyte apoptosis, and limiting tissue fibrosis [26,36]. These mechanisms include increased SIRT1 expression, decreased levels of acetylated p53 and Bax protein, and improved mitochondrial integrity [25,36]. Similar effects have also been observed in the case of cisplatin- and cyclophosphamide-induced toxicity [36].

Studies have also demonstrated the beneficial effects of resveratrol on the course of diabetic cardiomyopathy (DCM) and coronary artery disease (CAD) [35]. In type 2 diabetes models, resveratrol improved heart function by activating sirtuin 1 and AMPK kinase, significantly reduced oxidative-nitrative stress [26,32], and improved mitochondrial function [31]. In clinical trials in patients with type 2 diabetes and coronary artery disease, resveratrol supplementation modulated the expression of pro-inflammatory microRNAs, lowered systolic blood pressure and total cholesterol levels, although the effect on glycemic parameters was less consistent [24,27,33].

Resveratrol also exhibits strong anticoagulant and antiplatelet effects, mainly by inhibiting cyclooxygenase-1 (COX-1) activity, which contributes to a reduction in the synthesis of thromboxane A2 (TX A2), a powerful platelet aggregating factor. As a result, resveratrol may reduce the risk of blood clots and embolisms, contributing to an improvement in overall hemodynamic status [26,27,35]. However, it should be emphasised that despite the clear effects observed in preclinical studies, the results of clinical trials on hypertension and cardiovascular complications of diabetes remain inconsistent, which the authors attribute mainly to differences in dosage, duration of intervention and low bioavailability of resveratrol in humans [24,25,26,27,28,29,30,31,32,33,34,35]. It is worth noting that the authors of clinical studies also take into account a number of additional factors that differentiate the results, such as individual metabolism, intestinal microflora composition, gender, ethnicity of participants, patient selection and methods used to measure resveratrol concentration, which allows for a better interpretation of the discrepancies between preclinical and clinical effects [24,25,26,27,28,29,30,31,32,33,34,35,36,37].

### 5.4. Anticancer Activity

Resveratrol is a compound with broad therapeutic potential, and intensive research is being conducted on its chemopreventive and anticancer effects [38,39,40,41,42]. Resveratrol was chosen because of its ability to modulate numerous cell signaling pathways [41,42,43,44]. In the context of cancer, resveratrol is active against many types of cancer, affecting both the initiation and promotion stages of cancer transformation and the progression of the disease. These mechanisms include the induction of apoptosis, cell cycle arrest, inhibition of angiogenesis and metastasis, modulation of tumor metabolism, and effects on the tumor microenvironment [43,45,46]. Examples of cancers affected by resveratrol include hepatocellular carcinoma, gastric adenocarcinoma, squamous cell carcinoma of the oesophagus, lung adenocarcinoma, non-small cell lung cancer, endometrial cancer, cervical cancer, transitional cell carcinoma of the bladder, acute lymphoblastic leukaemia, chronic myeloid leukaemia, Burkitt’s lymphoma, multiple myeloma, Merkel cell carcinoma, glioblastoma multiforme, neuroblastoma, osteosarcoma, chondrosarcoma and others [38,39,40,41,42,43,44,45,46].

One of the most important effects of resveratrol in the context of cancer protection is the induction of apoptosis, or programmed cell death. It is a natural mechanism for eliminating damaged or mutated cells [38,39,42,47]. RSV activates the mitochondrial apoptosis pathway, leading to the loss of mitochondrial membrane potential [39,42,44,47], the release of cytochrome c, and the activation of caspases (proteolytic enzymes responsible for initiating and executing the apoptosis process). The ratio of proapoptotic Bax protein to antiapoptotic Bcl-2 also increases, leading to an imbalance in favor of cancer cell death [42,43,47,48]. Equally, resveratrol affects the regulation of the cell cycle by stopping the proliferation of cancer cells in specific phases, most often G1 or the transition from S to G2/M. This mechanism is associated with the activation of tumor suppressor genes, such as p53 and p21, which inhibit cyclins (including cyclins D1 and E) and cyclin-dependent kinases (CDKs) necessary for DNA replication and cell division [38,39,40,42,47].

Signal pathway modulation is another important aspect of resveratrol’s anti-cancer activity. This process is responsible for cell proliferation, survival, inflammation, and metabolism. Resveratrol affects a number of key signaling cascades, including PI3K/AKT/mTOR, which play a fundamental role in regulating the growth and survival of cancer cells. Inhibition of this pathway by resveratrol causes inhibition of proliferation, stimulation of autophagy and apoptosis, which has been observed in numerous models of prostate, ovarian and lung cancer [38,40,43,44,45,49]. Resveratrol also exhibits potent anti-inflammatory properties, mainly by blocking the activation of the transcription factor NF-κB. Inhibition of NF-κB leads to a decrease in the expression of genes encoding inflammatory mediators such as COX-2 (cyclooxygenase-2), TNF-α (tumor necrosis factor alpha) and IL-6 (interleukin 6), thereby reducing the inflammatory process that promotes cancer transformation and resistance to treatment [38,40,43,44].

Activation of the Nrf2 transcription factor is another important anti-cancer effect of resveratrol. It is responsible for inducing the expression of antioxidant and detoxification genes. Activation of this pathway contributes significantly to increasing cell resistance to oxidative stress and maintaining redox homeostasis, which in the context of chemoprevention acts as a protective measure against DNA damage [38,39,44]. However, it should be emphasized that this pathway can act in two ways, but its overactivation in some types of cancer may increase resistance to chemotherapy and radiotherapy [38,39]. In addition to the above-mentioned resveratrol, it also inhibits other pathways such as Wnt/β-catenin (important in colorectal cancer), STAT3 (involved in head and neck cancers and gliomas), Hedgehog (Hh, e.g., in prostate cancer), and MAPK/ERK1/2, responsible for growth signal transduction [42,43,49,90].

Resveratrol also exhibits anti-metastatic and antiangiogenic effects, significantly limiting the ability of cancer cells to invade and form distant metastases [43,44,90]. This substance has been shown to inhibit epithelial-mesenchymal transition (EMT), a process that is key to cancer cells acquiring invasive and migratory properties [40,90]. It also reduces the expression of matrix metalloproteinases (MMPs), especially MMP-9, which are responsible for the degradation of the extracellular matrix and facilitate the spread of cancer cells [38,42,43]. At the same time, resveratrol inhibits angiogenesis (the formation of new blood vessels in the tumor) [39,42,44] by inhibiting the expression of vascular endothelial growth factor (VEGF) and hypoxia-inducible factor (HIF-1α). The most important effect of these processes is the inhibition of tumor growth and development and the reduction in its ability to metastasize [39,40,42]. The anti-metastatic and anti-angiogenic effects of resveratrol have also been confirmed in animal models, where a reduction in the number of metastases and inhibition of angiogenesis have been observed. In clinical trials, efficacy is mainly limited by the low bioavailability of the compound, which requires the use of strategies to improve its delivery, such as nanoparticles or combination with other compounds that increase the concentration of the active form in the blood [38,39,42,43,44,49,90].

In terms of cancer cell metabolism, resveratrol affects their characteristic metabolic feature known as the Warburg effect. It involves the preferential use of aerobic glycolysis instead of oxidative phosphorylation even in the presence of oxygen [40,44]. Resveratrol can reverse this effect by interacting with glycolytic pathway enzymes such as hexokinase II and the pyruvate dehydrogenase complex [40]. Additionally, the modulation of these enzymes by resveratrol affects the sensitivity of cancer cells to chemotherapy, with tumour heterogeneity determining a varied response to these metabolic changes [40,44]. This leads to a slowdown in cancer metabolism and a reduction in the energy production necessary for rapid cancer cell growth. In HeLa cervical cancer cells, resveratrol has been shown to reduce the expression of proteins responsible for oxidative phosphorylation (OxPhos), leading to energy metabolism disruption and cell death [44].

Equally important is the effect of resveratrol on the tumor microenvironment (TME). It acts as a redox balance regulator, immunomodulator, and angiogenesis inhibitor [39]. Depending on its concentration, it can exhibit both antioxidant and pro-oxidative effects. In high doses, it generates oxidative stress leading to the death of cancer cells, while in healthy cells it has a protective function by neutralizing reactive oxygen species [38,39,44]. Resveratrol can also increase the cytotoxic activity of NK cells, which are responsible for destroying cancer cells, and modulate the macrophage population in the tumor microenvironment, transforming the tumor-promoting phenotype (M2) into an anti-tumor phenotype (M1) [39,91].

The efficacy of resveratrol has been confirmed in numerous in vitro and in vivo studies on various types of cancer. However, it should be emphasised that these effects depend on the type of cancer, the concentration of resveratrol and the duration of exposure. At low doses or with short exposure, the compound may have a protective or stimulating effect on proliferation, while at high doses and with longer treatment times, it induces apoptosis, stops the cell cycle and leads to epigenetic changes, which emphasises its hormetic nature [38,39,42,43,44,46,47,49,91]. In breast cancer, it exhibits cytotoxicity against MDA-MB-231 and 4T1 (human, invasive breast cancer, ER-negative), cell lines and increases their sensitivity to chemotherapy [38,42,44,46,47]. In colorectal cancer (CRC), it inhibits proliferation, induces apoptosis, and regulates the Wnt/β-catenin pathway [38,40,42]. In prostate cancer (androgen-dependent—LNCaP line and androgen-independent—PC3, DU145 lines), it limits proliferation and metastasis by influencing the Akt/microRNA-21 pathway [38,42,46,90]. It also acts in lung (Lung adenoma and non-small cell lung cancer), liver (HCC), and ovarian cancers (ovarian adenocarcinoma, serous ovarian cancer), where it induces apoptosis, autophagy, and modulates the expression of non-coding RNAs (miRNA, lncRNA) that affect metabolism and drug resistance [38,42,43,44,46,49].

### 5.5. Neuroprotection and Brain Protection

Due to its broad spectrum of biological activity, this compound attracts widespread interest among scientists. Resveratrol exhibits strong neuroprotective effects, which are being intensively studied in terms of counteracting neurodegenerative diseases such as Alzheimer’s disease (AD) and Parkinson’s disease (PD) [92,93]. The protective mechanisms of resveratrol are complex and include, among others, the alleviation of oxidative stress, inhibition of neuroinflammation, prevention of apoptosis, and modulation of numerous signalling pathways responsible for cell survival [93,94,95,96].

One of the key aspects of resveratrol’s neuroprotective effect is its ability to neutralise oxidative stress and stabilise mitochondrial function. High concentrations of reactive oxygen species play an important role in the development of both Alzheimer’s and Parkinson’s disease, as well as damaging the cellular structures of neurons. This polyphenol effectively eliminates harmful reactive oxygen species and free radicals, thus protecting cells from peroxidation damage to membranes [92,93,94,96,97,98]. The expression of key antioxidant enzymes such as superoxide dismutase (Mn-SOD), glutathione peroxidase (GPx) and glutathione (GSH) is also activated, leading to an increase in the brain’s natural oxidative defence. In addition, resveratrol activates the Nef2 transcription factor, which induces the expression of heme oxygenase 1 (HO-1). This enzyme is key to protecting neurons from degradation by oxidative stress [93,94,95,96,97,98]. At the mitochondrial level, this compound prevents their dysfunction by maintaining the proper expression of genes responsible for fusion and division processes (including Mfn2—Mitofusin-2, Opa1—Optic Atrophy Protein 1, Drp1—Dynamin-Related Protein 1, Fis1—Fission Protein 1), which protects against β-amyloid (Aβ) toxicity [98]. In addition, it is responsible for inhibiting quinone reductase (QR2) activity, whose overexpression in the hippocampus is associated with severe memory impairment and excessive oxidative stress [99].

Resveratrol, through its strong anti-apoptotic effect, influences the regulation of neuronal survival mechanisms [95]. Under conditions of severe oxidative stress or exposure to Aβ, it protects nerve cells from programmed cell death [92]. A key element of this mechanism of action is the modification of the Bcl-2/Bax protein ratio, where an increase in the level of anti-apoptotic Bcl-2 and a decrease in pro-apoptotic Bax lead to the inhibition of caspase-3 activation, i.e., the main enzyme initiating apoptosis [93,94,95,96,99]. In addition, resveratrol stimulates the expression of neurotrophins such as BDNF (brain-derived neurotrophic factor) and GDNF (glial-derived neurotrophic factor), which promotes the regeneration and survival of dopaminergic neurons [93,94,97,100].

At the molecular level, resveratrol acts mainly through the activation of two main regulatory enzymes, SIRT1 (Sirtuin 1) and AMPK (AMP-activated protein kinase). SIRT1 is responsible for the deacetylation of proteins such as p53 (p53 tumour suppressor protein), NF-κB (nuclear factor κB—activator of light chain immunoglobulins in activated B lymphocytes) and PGC-1α (coactivator 1 of the peroxisome proliferator-activated receptor gamma, alpha), which leads to the inhibition of inflammatory processes, improvement of energy metabolism processes and increased resistance of neurons to oxidative stress. AMPK, on the other hand, is a key factor in maintaining cellular energy homeostasis, and its activation by resveratrol further enhances the action of SIRT1 by increasing NAD+ (nicotinamide adenine dinucleotide) levels [92,94,98,99,101]. The presence of both these pathways contributes to the induction of autophagy, i.e., the process of removing damaged mitochondria and protein aggregates, which is particularly important in preventing neurodegenerative processes [93,101]. Furthermore, resveratrol has been shown to improve proteostasis, thereby increasing the activity of the 20S proteasome and the degradation of abnormal proteins, which counteracts their accumulation in neurons [102]. However, it should be noted that most of these observations come from preclinical studies and are mainly described in qualitative terms, without systematic analysis of the relationship between the effect and the dose and route of administration of resveratrol [92,93,94,98,99,101,102].

In Alzheimer’s disease, resveratrol exhibits multidimensional neuroprotective effects. Among other things, it regulates β-amyloid metabolism by reducing the activity of β-secretase (BACE1) while increasing the expression of neprilysin, an enzyme that degrades amyloid. It also promotes the removal of toxic Aβ oligomers [92,94,99,102]. It alleviates tau protein pathology by limiting its phosphorylation and acetylation, which enables its degradation by the ubiquitin-proteasome system. This directly prevents the formation of neurofibrillary tangles [93,94,102]. This polyphenol also significantly strengthens the blood-brain barrier by reducing the level of MMP-p metalloproteinase in the cerebrospinal fluid [92,94,95,97,101]. Studies conducted on AD animal models (including 3xTg-AD (tri-transgenic mouse model of Alzheimer’s disease)) have shown that resveratrol supplementation protects against memory loss and significantly improves cognitive function [102]. At the same time, it should be noted that the efficacy of resveratrol in humans is limited mainly by its low bioavailability and rapid metabolism, which makes it difficult to extrapolate results from animal models to patients [92,93,94,95,97,99,101,102].

In Parkinson’s disease, resveratrol protects dopaminergic neurons from oxidative stress and dopamine-induced apoptosis. This mechanism involves the activation of AMPK (AMP-activated protein kinase) and significant inhibition of COX-2 (cyclooxygenase-2). Studies conducted on model organisms of MPTP-induced Parkinson’s disease showed that the use of resveratrol nanoparticles had a significantly stronger protective effect, which clearly suggests the high therapeutic potential of this form in the future. However, it should be emphasised that these are preclinical results, and the potential clinical application of resveratrol nanoparticles is currently limited by, among other things, the lack of clinical trials and challenges related to the safety and standardisation of nanocarrier systems [92].

In cases of acute central nervous system damage, such as ischaemic stroke or traumatic brain injury (TBI), resveratrol also exhibits significant neuroprotective activity. In the case of stroke, it reduces infarct volume, limits neuroinflammation and oxidative stress, and additionally protects the integrity of the BBB (blood-brain barrier) by regulating the expression of SUR1 (sulfonylurea receptor 1), AQP4 (aquaporin-4) and MMP-9 (Matrix Metalloproteinase 9) [95,101,103]. In TBI models, it reduces brain oedema, alleviates behavioural disorders and reduces the activity of the NLRP3 inflammasome (NOD-like receptor with pyrin domain 3) and the TLR4/NF-κB pathway (Toll-like receptor 4/Nuclear factor κB—activator of the immunoglobulin light chain in B lymphocytes), while effectively preventing neuronal apoptosis [96].

### 5.6. Hepatoprotective Action/Liver Protection

One of the main factors causing hepatocyte damage is oxidative stress, which plays a key role in the pathogenesis of liver diseases such as MASLD (metabolic dysfunction-associated steatotic liver disease) [104]. Resveratrol has a very strong antioxidant effect, consisting in the naturalisation of reactive oxygen species (ROS) and preventing lipid peroxidation [38,105,106,107,108]. Studies have shown that resveratrol reduces the concentration of malondialdehyde (MDA) and nitric oxide (NO) in liver tissues, while increasing the activity of antioxidant enzymes such as superoxide dismutase (SOD) and catalase (CAT) [38,104,105,109]. In addition, it activates the Nrf2 transcription factor, which initiates the expression of genes responsible for encoding defence enzymes such as heme oxygenase-1 (HO-1) and glutathione S-transferase. Activation of this pathway prevents mitochondrial dysfunction and peroxidation damage to cell membranes, which significantly supports the regeneration and survival of hepatocytes [104,107,108,109].

Chronic inflammation is a key factor in the progression of liver damage, leading to fibrosis and cirrhosis [108]. Resveratrol exhibits potent anti-inflammatory effects by inhibiting the NF-κB signalling pathway, the main regulator of pro-inflammatory gene expression [16,38,104,107,108,109]. Blocking NF-κB activation limits the synthesis of cytokines such as TNF-α, IL-6 and IL-1β, thereby reducing the infiltration of inflammatory cells in the liver. Resveratrol also inhibits the expression of cyclooxygenase-2 (COX-2) and iNOS, limiting the production of prostaglandins and nitric oxide, which sustain inflammation. As a result, the chronic immune response is inhibited and tissue integrity is improved [38,104,105,106,108,109].

Non-alcoholic fatty liver disease (NAFLD/MASLD) is currently one of the most common metabolic disorders in which resveratrol has a particularly beneficial effect [104,105,107]. It activates the AMPK/SIRT1 (AMP-activated protein kinase/Sirtuin 1) signalling pathway, which is responsible for increasing fatty acid β-oxidation and inhibiting de novo lipogenesis. AMPK activation leads to the phosphorylation and inactivation of key lipogenic enzymes such as ACC (acetyl-CoA carboxylase), which limits the accumulation of triglycerides in hepatocytes [104,105,107,109]. Resveratrol also promotes the induction of autophagy, and in particular lipophagy, the process of degradation of excess lipid droplets [104,107,108]. Thanks to this action, resveratrol prevents fatty liver disease, improves the lipid profile, thereby lowering total cholesterol, LDL and triglyceride levels, while increasing the proportion of HDL [38,104,105]. At the same time, it should be noted that most of these effects have been clearly confirmed in animal models, while clinical studies in humans show less consistent results, which is mainly due to the low bioavailability of resveratrol, its rapid metabolism and high individual variability [38,104,105,107,109].

Progressive fibrosis of the liver is an important step in the transition to cirrhosis and hepatocellular carcinoma [38,105,109]. Resveratrol limits the activation of hepatic stellate cells (HSCs), which are responsible for the excessive production of collagen and extracellular matrix components. Studies in animal models have shown that resveratrol administration reduces collagen fibre accumulation and TGF-β1 expression, a major pro-fibrotic factor [109]. At the same time, resveratrol protects hepatocytes from apoptosis by inhibiting caspase-3 activity and modulating the Bcl-2/Bax protein ratio. This action promotes the preservation of the normal structure of the liver parenchyma and limits the progression of degenerative changes [105,106,108,110].

Resveratrol is also highly effective in many experimental models of liver damage. In cases of drug toxicity, e.g., caused by paracetamol [105,106] or zinc oxide nanoparticles (ZnO NPs) [110], resveratrol significantly reduces the levels of hepatocyte damage markers such as cytochrome P450 (CYP2C11) [106]. It also protects against damage caused by ischaemia-reperfusion (I-R) [38,105] and oxidative stress accompanying haemorrhagic shock [111]. In this case, the hepatoprotective effect of resveratrol is mediated by oestrogen receptors (ER), suggesting that this mechanism is dependent on hormonal signalling [111].

### 5.7. Impact on Gut Microbiota and Gut Metabolites

Resveratrol has a strong ability to restore microbial balance in the gut by correcting dysbiotic disorders [50,107]. Preclinical studies indicate that resveratrol increases the proportion of *Bacteroidetes* and *Actinobacteria*, while reducing the level of *Firmicutes*. This change is metabolically significant, as a high *Firmicutes/Bacteroidetes* ratio is considered a biomarker of obesity and insulin resistance. Resveratrol therefore acts as a counteracting factor to these adverse changes by modulating the components of the microbiota. At the family and genus level, resveratrol enriches the flora with probiotic bacteria such as Bifidobacteriaceae and Lactobacillus, while inhibiting the growth of potentially pathogenic microorganisms such as Enterobacter and Streptococcus. Such selective modulation supports the intestinal barrier, reduces the translocation of endotoxins (e.g., LPS—lipopolysaccharides) and reduces local and systemic inflammation [50,51].

One of the key mechanisms through which resveratrol affects the microbiota is its influence on the profile of metabolites produced by intestinal bacteria. Resveratrol modulates the enzymatic activity of the microflora, leading to changes in the concentration of aromatic amino acid derivatives (e.g., tryptophan, tyrosine, phenylalanine). These metabolites play an important role in communication between the microbiota and the host, influencing, among other things, inflammation, intestinal barrier integrity and lipid metabolism [112]. It has been shown that resveratrol, through its effect on the intestinal flora, modifies the expression of lipogenic genes such as Gpat1 (glycerol-3-phosphate acyltransferase 1), Mogat (monoacylglycerol O-acyltransferase) and Pparg (peroxisome proliferator-activated receptor gamma (PPAR-γ)), which translates into reduced triglyceride synthesis and improved lipid profiles. This means that the metabolic effects of resveratrol are not only the result of its direct action in tissues, but also a consequence of its interaction with the gut microbiota and its metabolites [50].

As a result of microbiological changes in the intestines, resveratrol is converted to 3-hydroxyphenylpropionic acid and 4-hydroxyphenylpropionic acid, which have their own, often stronger biological effects. Studies conducted on animal models with metabolic dysfunction-associated steatotic liver disease (MASLD) showed that administration of metabolites (100 µM for 13 weeks) reduced body weight and liver weight, improved liver histology, and reduced systemic inflammation. Importantly, these metabolites had a stronger effect on the expression of genes regulating cholesterol metabolism than unchanged resveratrol itself. This means that its microbiological transformations not only do not weaken its biological activity, but may even increase its effectiveness through the formation of new, active compounds with metabolically beneficial effects. This analysis suggests that microbiological transformations of resveratrol may significantly modify its functional bioavailability, and that the observed therapeutic effects in vivo may result largely from the activity of its metabolites rather than the parent compound itself [107,113].

The accumulated data indicate that the gut-liver axis plays a key role in the mechanism of action of resveratrol. By modulating the composition of the microbiota and its metabolites, resveratrol indirectly influences the processes occurring in the liver, including lipogenesis, fatty acid oxidation and inflammatory processes. The regulation of the gut microbiota and the conversion of resveratrol to active phenolic metabolites contribute to improved liver function, lower plasma lipid levels and reduced oxidative stress. In this way, resveratrol acts as a link between the microbiological and metabolic aspects of health, and its action can be described as a holistic tuning of the intestinal and hepatic ecosystems [107,112].

## 6. Discussion

Resveratrol is a natural polyphenol with a broad spectrum of biological activity, including anti-inflammatory, antioxidant, cardioprotective, neuroprotective and anti-cancer properties. Its action is mainly due to the modulation of key cell signalling pathways such as NF-κB, SIRT1, AMPK and Nrf2, which play an important role in the regulation of inflammatory processes, redox balance and energy metabolism. Numerous preclinical and clinical studies indicate the beneficial effects of resveratrol on the cardiovascular, immune and nervous systems, making it one of the best-known phytochemicals with therapeutic potential.

Despite its promising biological properties, the practical application of resveratrol is limited by its low bioavailability, resulting from limited water solubility, chemical instability and intense hepatic metabolism. In response to these limitations, numerous strategies are being developed to improve bioavailability, including the use of nanotechnology, lipid systems, cyclodextrin complexes, micellar formulations, and synergistic combination with metabolism-inhibiting compounds such as piperine. These solutions show potential for increasing plasma resveratrol concentrations and prolonging its duration of action.

## 7. Conclusions

Based on the analysis of available data, it can be concluded that resveratrol exhibits a broad spectrum of biological activity and significant therapeutic potential in the prevention and treatment of many chronic diseases, including cardiovascular diseases, cancer, metabolic disorders and neurodegenerative diseases. Its action results from the modulation of key cell signalling pathways and its ability to regulate inflammatory and oxidative processes.

At the same time, a major limitation to the use of resveratrol in clinical practice remains its low bioavailability, resulting from limited water solubility, chemical instability and intensive first-pass metabolism in the liver. These factors significantly limit the concentrations of the compound achieved in plasma and target tissues, which may affect the efficacy of the observed biological effects in vivo.

Despite the dynamic development of strategies to increase the bioavailability of resveratrol, such as nanotechnology, lipid systems, cyclodextrin complexes, and co-administration of metabolic inhibitors, most of the available data comes from preclinical studies or short-term human studies. However, there is a lack of long-term, well-designed clinical trials that would unequivocally confirm the therapeutic efficacy and safety of these solutions.

Therefore, future studies should focus on the clinical evaluation of optimised forms of resveratrol, the determination of optimal doses and administration regimens, and long-term monitoring of the safety of the therapy. Only such an approach will allow the potential of resveratrol to be fully exploited as a component of modern preventive and therapeutic strategies.

## Data Availability

Data sharing does not apply in this article as no datasets have been generated.
